# Identification of the target and mode of action for the prokaryotic nucleotide excision repair inhibitor ATBC

**DOI:** 10.1042/BSR20220403

**Published:** 2022-05-30

**Authors:** Lorenzo Bernacchia, Antoine Paris, Arya Gupta, Alexandra A. Moores, Neil M. Kad

**Affiliations:** School of Biological Sciences, University of Kent, Canterbury CT2 7NH, U.K.

**Keywords:** DNA synthesis and repair, inhibitor, multidrug resistance

## Abstract

In bacteria, nucleotide excision repair (NER) plays a major role in repairing DNA damage from a wide variety of sources. Therefore, its inhibition offers potential to develop a new antibacterial in combination with adjuvants, such as UV light. To date, only one known chemical inhibitor of NER is 2-(5-amino-1,3,4-thiadiazol-2-yl)benzo(f)chromen-3-one (ATBC) exists and targets *Mycobacterium tuberculosis* NER. To enable the design of future drugs, we need to understand its mechanism of action. To determine the mechanism of action, we used *in silico* structure-based prediction, which identified the ATP-binding pocket of *Escherichia coli* UvrA as a probable target. Growth studies in *E. coli* showed it was nontoxic alone, but able to impair growth when combined with DNA-damaging agents, and as we predicted, it reduced by an approximately 70% UvrA’s ATPase rate. Since UvrA’s ATPase activity is necessary for effective DNA binding, we used single-molecule microscopy to directly observe DNA association. We measured an approximately sevenfold reduction in UvrA molecules binding to a single molecule of dsDNA suspended between optically trapped beads. These data provide a clear mechanism of action for ATBC, and show that targeting UvrA’s ATPase pocket is effective and ATBC provides an excellent framework for the derivation of more soluble inhibitors that can be tested for activity.

## Introduction

The development of new targets that inhibit bacterial survival is critical to the discovery of new antimicrobial drugs. Numerous pathways have been targeted including cell wall synthesis, protein synthesis, nucleic acid synthesis, and general metabolism. However, the development of new antibiotic classes has been slow, and despite the identification of new antimicrobial targets, no new classes of compounds have been identified since 1987 [1]. Antimicrobial resistance (AMR) is an on-going complication in the use of antimicrobials, reducing the effectiveness of current treatments, and further exacerbating the need for the development of new drugs and targets. AMR represents a heavy burden on both, life, and the economy with an estimation of 10 million deaths per year by 2050 [2]. Additionally, with pharmaceutical companies changing their focus from antimicrobial drug discovery, there is an urgent need to find novel compounds to treat infections [3,4]. Some of these compounds could be effective as combination therapies, or adjuvants.

One area of exploitation that could potentially represent a new target class is nucleotide excision repair (NER). This DNA repair system is present in both eukaryotic and prokaryotic cells, but substantially distinct at the molecular level [5], making it an ideal target. Its high level of conservation among bacteria also raises the prospect for the development of a widely deployable antibiotic. NER detects and repairs a wide variety of DNA lesions making it essential for cell survival when exposed to certain DNA-damaging agents, including exposure to exogenous agents such as UV irradiation, toxins, and endogenous metabolic by-products [6,7]. NER is a multienzyme cascade initiated by UvrA, a homodimeric molecule with four ATP-binding sites. Each monomer possesses two distinct ATPase cassettes, one toward the C-terminus (distal) and the other toward the N-terminus (proximal). The precise role of the ATPases is still not certain, but they interact in a negatively co-operative manner, such that the distal ATPase is necessary for DNA binding and damage recognition, which activates the proximal ATPase and allows the formation of a complex with the second protein in the system, UvrB [8–10]. Once UvrB confirms the presence of damage it recruits UvrC and other downstream factors to repair the lesion [11].

**Figure 1:**
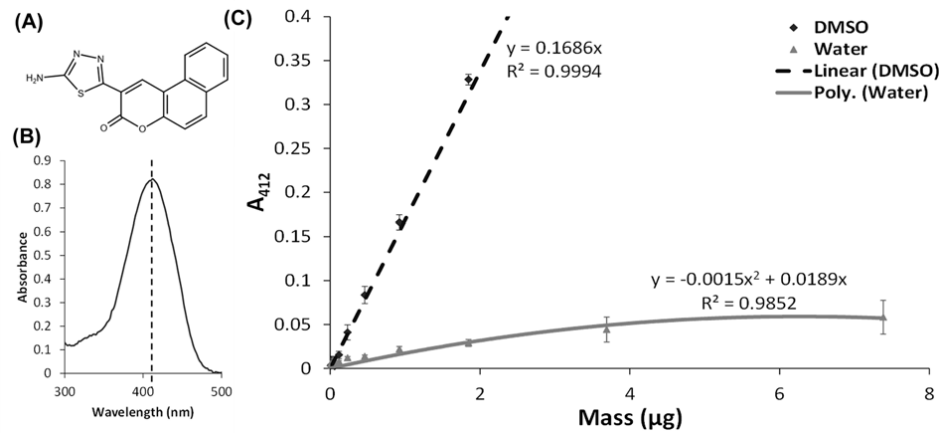
Assessing ATBC solubility in DMSO and water (**A**) ATBC structure, PubChem CID = 399112. (**B**) Absorbance spectrum of 100 µM ATBC showing a peak at 412 nm. (**C**) ATBC solubility profile, the difference in solubility between DMSO and water is approximately ten-times greater, shown by the difference in slope of the linear part of the two trend lines. The solubility profile was repeated three-times and the error bars represent the standard error of the mean.

The repair of DNA damage is crucial to the survival of all cells; therefore, NER is involved in repairing lesions produced from UV, chemical exposure or during host immune defense due to reactive oxygen and nitrogen species being produced [12,13]. Therefore, inhibiting this repair mechanism presents a potentially interesting pharmaceutical target. The only known inhibitor of prokaryotic (*Mycobacterium tuberculosis*) NER is 2-(5-amino-1,3,4-thiadiazol-2-yl)benzo[f]chromen-3-one (ATBC, Figure 1A), and was found in a 38000 molecule drug screen [14], however, the precise mechanism of action or target was not identified.

Here, we set out to understand how ATBC functions, as a starting point for assessing its promise as a novel antimicrobial agent. By subjecting *Escherichia coli* to UV radiation and 4-nitroquinoline 1-oxide (4-NQO), both DNA-damaging agents specific to NER, we confirm ATBC is effective [14]. However, to determine ATBC’s mechanism of action, we used an *in silico* docking approach that predicted a strong interaction with UvrA’s ATPase site. This interaction was confirmed by directly measuring the ATPase of purified UvrA *in vitro*. To understand how ATBC binding to UvrA’s ATPase site affects its function we also performed a single-molecule imaging assay to reveal that ATBC reduces the binding of UvrA to DNA. Although these studies reveal that ATBC could be used as a potential antimicrobial, we also found ATBC is a substrate of the multidrug efflux pump TolC. In addition, ATBC is highly insoluble at the concentrations required for effective inhibition. Therefore, we conclude that ATBC binds to UvrA’s ATP pocket, disturbs the allosteric communication within the protein, and prevents binding to DNA. However, the physical properties of ATBC likely prevent its widespread use. Nonetheless, our results show that the use of *in silico* docking provides good prediction for mechanism of action and that inhibitors of UvrA’s ATPase are an excellent target for a potential novel class of antimicrobial.

## Materials and methods

### Bacterial strains and plasmids

Strains used in the study included *E. coli* MG1655, MG1655 Δ*tolC*, MG1655 Δ*tolC* Δ*uvrA*, and BL21 Δ*uvrA* Δ*uvrB*. The mutant strains were generated by P1 transduction of the respective gene knockouts from the Keio collection [15]. The double knockout strains (e.g., BL21 Δ*uvrA* Δ*uvrB*), were generated sequentially, and the plasmid pCP20 was used to eliminate the antibiotic resistance marker following the first transduction [16]. First, *uvrA* deletion from the Keio collection was used to transduce BL21 to produce BL21 Δ*uvrA* (Kan^R^). The kanamycin resistance gene was subsequently eliminated using pCP20 as described above [17] to produce BL21 Δ*uvrA*. The resultant strain was then transduced with the *uvrB* deletion to generate BL21 Δ*uvrA* Δ*uvrB* (Kan^R^), which was used for protein expression and purification. The same approach was used to generate the mutant MG1655 Δ*tolC* Δ*uvrA.* All strains were stored at -80°C and grown at 37°C with aeration when required, with antibiotic selection if appropriate.

### Media and compounds

Bacterial cultures were grown overnight prior to the experiment in LB Broth, Miller (ThermoFisher). Survival assays were performed using MOPS Minimal Medium [18] supplemented with 0.2% glucose. ATBC (Green Pharma, Ambinter) and 4-NQO (Merck) were dissolved in 100% DMSO at stock concentrations of 5 and 20 mM, respectively, and stored at -20°C.

### Solubility of ATBC

ATBC was dissolved as a 5 mM stock solution in DMSO. Twofold serial dilutions were prepared in microcentrifuge tubes using DMSO or water as the solvent. After extensively vortex-mixing, the solution was centrifuged at 13000 rpm for 30 min to pellet precipitated compound. One hundred microliters of the supernatant were added to a microtiter plate and the OD_412_ was recorded in a plate reader (id5 Spectramax, Molecular Devices). Experiments were performed in triplicate.

### Protein purification

Unlabeled UvrA (gene obtained from NIG, Kyoto, Japan) was engineered with a C-terminal flexible linker followed by an AviTag, Tev protease site, and His tag for purification. A fluorescently labeled version was also made by engineering mNeonGreen to the C-terminal prior to the His tag. All constructs were cloned into an IPTG inducible vector (pJB). Cultures were induced at an OD_600_ of 0.4–0.6 with 0.5 mM IPTG and grown at 18°C overnight. Cells were pelleted and resuspended in 10 mM Tris–HCl (pH 8), 1 mM DTT, 500 mM NaCl with protease inhibitor cocktail ((no EDTA) Thermo Fisher scientific)), and 1 mM phenylmethylsulfonyl fluoride (PMSF). Cells were subsequently lysed with 100 µg/ml lysozyme, followed by sonication, and then centrifugation at 18000 rpm. The supernatant was loaded onto a Proteus ‘1-step batch’ midi plus spin column (Protein Ark column) with Ni-NTA resin (Thermo Scientific™ HisPur™) pre-equilibrated with 50 mM imidazole, 500 mM NaCl, 50 mM sodium phosphate (pH 7.5), and purified with increasing concentrations of imidazole. The protein was stored at -20°C in 50 mM Tris (pH 7.5), 500 mM KCl, 0.1 mM EDTA, 5 mM DTT, 50% glycerol, and concentration was estimated measuring the OD_280_.

For single-molecule experiments, UvrA-mNeonGreen was induced at 37°C for 3 h and cells harvested and resuspended in a low imidazole buffer (50 mM NaPO_4_ (pH 8), 14 mM imidazole, 300 mM NaCl) with 1 mM PMSF. The cells were lysed by sonication, centrifuged, and the supernatant was filtered prior to protein concentration determined mNeonGreen absorbance at OD_506_. The lysate was aliquoted, flash frozen in liquid nitrogen, and stored at -80°C.

### Survival assays

Cell survival assays were performed based on the Clinical and Laboratory Standards Institute (CLSI) guidelines [19]. Bacteria were inoculated from a frozen glycerol stock in sterile LB broth (supplemented with kanamycin if required) and grown until exponential phase. The bacteria were then diluted in sterile MOPS Minimal Medium [18] supplemented with 0.2% glucose. ATBC was used at the concentrations stated, but the final concentration of DMSO did not exceed 2.5% v/v to prevent interference with bacterial growth. Plates were then incubated at 37°C while shaking in a plate reader (id5 Spectramax, Molecular Devices), and OD_600_ readings were taken every 30 min. Three biological replicates with technical repeats were performed for each assay, and the error was reported as the standard error of the mean. To visualize the results we used resazurin, a blue compound that is irreversibly metabolized by living cells to the pink-colored product resorufin [20]. Following incubation, 50 µl of buffered resazurin (0.3 mg/ml resazurin sodium salt, 400 mM MOPS, 40 mM Tricine, pH 7.4) was added to the plate and imaged after a further 4 h incubation at 37°C. For UV experiments, the cells were irradiated with 75 J/m^2^ UV at 254 nm using a UV cross-linker (UVP/Analytik Jena UV Crosslinker CX-2000) before transferring the bacteria to the microplate.

### *In silico* docking

Computational substrate docking was performed using the open-access software AutoDock Vina [21]. The *E. coli* UvrA structure used was derived from the AlphaFold Protein Structure Database [22]. All 3D structures of the compounds were downloaded from the PubChem [23] and then prepared for docking by energy minimization using the OpenBabel [24]. The MMFF94 force field was used for energy minimization, as suggested in Open Babel’s documentation [25]. The protein was converted into pdbqt format using AutoDock tools 1.5.7 [26], after polar hydrogens were added. For docking with AutoDock Vina, a search space including the entire protein was defined to allow the algorithm to probe all surfaces. As a control, ATP was docked alongside ATBC. The docking was repeated six-times with each molecule and the results analyzed and imaged using the PyMOL.

### NADH-linked ATPase assay

0.5 mM Phosphoenolpyruvate was prepared in ABC buffer (50 mM Tris–HCl (pH 7.5), 50 mM KCl, 10 mM MgCl_2_), aliquoted, flash-frozen in liquid N_2_, and stored at -20°C. All the components were added sequentially at the following final concentrations: 1 mM DTT, 210 µM NADH, 20 µl of pyruvate kinase (600–1000 U/ml), and lactate dehydrogenase (900–1400 U/ml, premixed stock from Merck) per 1 ml. The slope of the linear decrease in OD_340_ due to NADH consumption was used to determine UvrA *k_cat_* values. UvrA was incubated for approximately 5 min with 50 µM ATBC [14], prior to starting the reaction with the addition of 1 mM ATP. Controls (without ATBC) were also incubated for 5 min prior to the addition of ATP. All reactions were repeated multiple times, and the error was reported as the standard error of the mean.

### Single-molecule imaging on suspended DNA filaments

UvrA-mNeonGreen binding to DNA was imaged using a C-trap system (Lumicks, Netherlands). This system enables the tethering of individual 3′ biotin-tagged bacteriophage lambda DNA molecules (48.5 kbp or 16 µm contour length) between streptavidin-coated beads held in optical traps. Protein binding was imaged 10 µm above the surface with the TIRF objective and a 488-nm laser at 30% power with the angle of incidence altered to enable far-field imaging [27]. Videos were acquired with exposure synchronization (4.3 Hz, 200 ms exposure) for 200 frames. UvrA-mNeonGreen containing cell lysate was diluted in ABC buffer to a final concentration of 5 nM, and when used, ATBC was added to a final concentration of 50 µM. To allow complete decoration of the protein to the tethered DNA, the beads and DNA tether were moved into a microfluidic channel containing UvrA-mNeonGreen with or without ATBC under lateral flow at 0.1 bar for 2 min. After this time, the beads and tether were then rapidly moved to a protein and ATBC-free channel for observation. During this workflow, the DNA tension is constantly monitored and adjusted to 50 pN. For quantification of binding events, the beads and DNA tether were moved into a microfluidic channel containing UvrA-mNeonGreen with or without ATBC. This was done in parallel with UvrA-mNeonGreen samples, prepared at the same time, being flowed into the sample chamber. DNA tension was raised to 50 pN and after flow of protein at a pressure of 0.2 bar for 2 min, flow was stopped, and images were taken in the protein-containing channel and not moved into a separate channel.

## Results

### Determining ATBC solubility in aqueous solution

To test the solubility of ATBC, we utilized a simple assay where the compound was solubilized in DMSO or water at decreasing concentrations, and after vigorous mixing, centrifuged to remove any precipitates. Since ATBC absorbs in the visible spectrum with a peak at 412 nm (Figure 1B), we were able to accurately determine the amount left in solution. With a computed log P of 2.9 [28], ATBC shows good solubility in DMSO, with a solution concentration directly proportional to the mass of added compound. When dissolved in water, precipitation was immediately visible even in the tip used for pipetting. The solution concentration of ATBC was found to be significantly lower in water and did not follow a linear trendline, indicating solution saturation within the measured range (Figure 1C). Furthermore, at low added masses of ATBC, the linear slopes for water versus DMSO are much lower also indicating reduced solubility.

### Evaluating the effects of DNA-damaging agents on NER active and inactive cells

**Figure 2:**
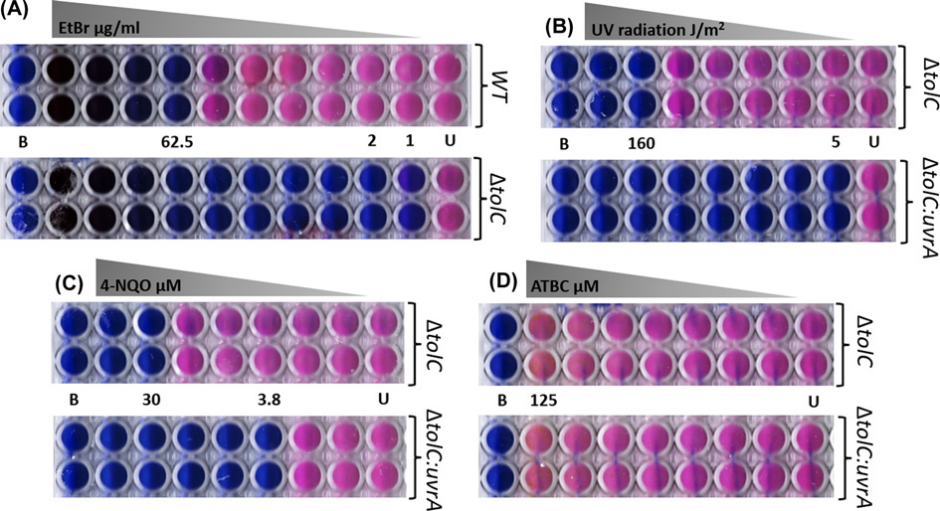
Determining MIC After 20 h incubation of cells at 37°C (16 h incubation prior to the addition of resazurin and further incubation for 4 h), the blue color shows no growth, whereas the pink wells represent growing cells (B = MOPS minimal media as sterility control, U = untreated cells). Numbers refer to concentrations or exposure as indicated. (**A**) The EtBr MIC shows a ≈64-fold increase in sensitivity when the TolC pump is knocked out. (**B**) UV irradiation MIC shows a ≈32-fold increase in sensitivity to UV with the uvrA knockout. (**C**) 4-NQO MIC shows a 30-µM MIC for the compound, which decreased eightfold in the uvrA knockout, confirming the compound to be a substrate for NER. (**D**) ATBC MIC, the MIC is > 125 µM for both strains used, indicating ATBC is nontoxic. The experiments were performed in triplicate on three independent days. No appreciable difference was identified among the replicates. A black and white version of this figure has been placed in the Supplementary material for improved visual accessibility (Supplementary Figure S1).

NER is the primary mechanism used by *E. coli* to repair damage caused by the UV exposure [29]. In addition, 4-NQO has been shown to elicit similar repair responses [30,31] and therefore, we set out to establish the extent of damage that *E. coli* can tolerate. To enhance the potential efficacy of compounds, we also created an efflux pump knockout Δ*tolC*. As a test for the effectiveness of this mutant strain, we determined the minimum inhibitory concentration (MIC) for ethidium bromide, a known substrate of TolC [32], relative to wild type. Figure 2A shows a 64-fold reduction in the MIC for Δ*tolC* from 62.5 µg/ml to approximately 1 µg/ml; validating the effect of this knockout. UV damage generates base adducts targeted by NER [11,29]; therefore, in the absence of NER one would expect a considerably lower tolerance for UV exposure. Comparing the tolerance to UV exposure of Δ*tolC* and Δ*tolC:uvrA*, a strain with UvrA knocked out to interrupt NER shows that while Δ*tolC* tolerates ∼160 J/m^2^ UV exposure, the *uvrA* mutant has <5 J/m^2^ UV tolerance (Figure 2B). 4-NQO forms adducts on DNA following metabolic conversion to 4-hydroxyaminoquinolone 1-oxide, mimicking the effects of UV exposure [7,30,31]. We found the MIC for 4-NQO of 30 µM in MG1655 Δ*tolC*, decreased eightfold to 3.8 µM in the double mutant (Figure 2C), confirming the resolution of these adducts using NER. Finally, we determined that ATBC did not impair bacterial growth even at the highest concentrations useable in the study for WT (MIC > 125 µM, Figure 2D) or Δ*tolC:uvrA*. These concentrations are masked by the poor solubility of this compound (see above), making estimates of the actual concentration unreliable.

### ATBC is effective at impairing NER

**Figure 3:**
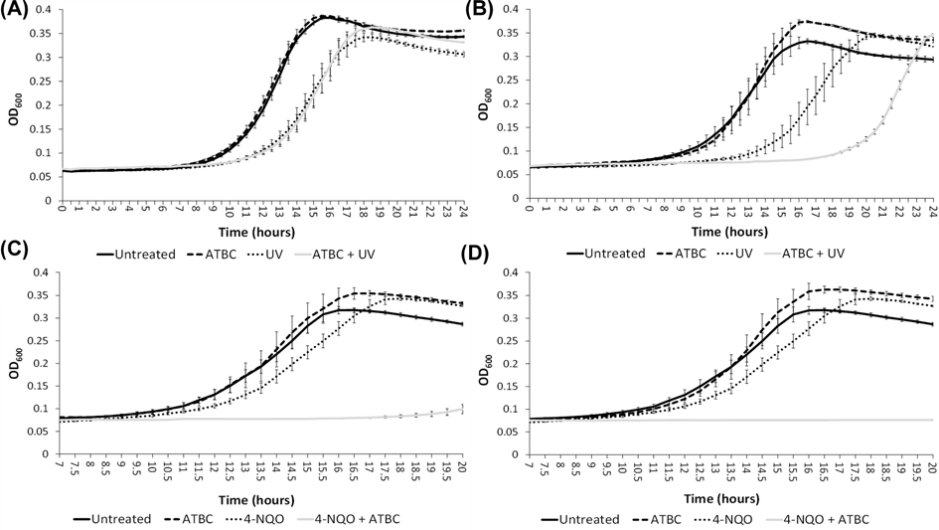
Effect of ATBC on MG1655 and MG1655 ΔtolC bacterial growth after exposure to 254 nm UV radiation Growth curves measured in a plate reader show (**A**) MG1655 growth in presence and absence of ATBC 5 µM with and without 75 J/m^2^ of 254 nm UV radiation. A shift in mid-exponential growth can be observed between the UV-treated sample and the UV untreated, the cells treated with ATBC show the same growth profile. (**B**) MG1655 ΔtolC growth in the presence and absence of ATBC 5 µM with and without 75 J/m^2^ UV radiation. A shift in mid-exponential growth is also observed between the UV-treated sample and the UV-untreated sample and an additional shift in the mid-exponential appears as the result of the combinatorial effect of UV and ATBC. (**C**) MG1655 ΔtolC growth in the presence and absence of 5 µM ATBC with DNA damaged by 10 µM 4-NQO. The combinatorial effect can be seen as a significant inhibition of growth and (**D**) as complete lack of growth when cells were treated with 10 µM ATBC. The experiments were done in triplicate on three independent days (the error bars represent the standard error of the mean, *n* = 3).

To evaluate whether ATBC is an effective antagonist of NER, we irradiated *E. coli* with a sublethal UV intensity (75 J/m^2^), which resulted in an ∼3 h delay in reaching the mid exponential point of growth (Figure 3A, compare solid black and dotted lines). When cells were treated with ATBC (5 µM), we did not observe any delay in the midpoint of growth compared with UV-only treated cells (Figure 3A, compare solid black and dashed lines). These observations were also true for the Δ*tolC* mutant (Figure 3B, compare solid black and dashed lines). However, we observed a substantial delay to growth by ∼5 h when the cells were treated with 5 µM ATBC and 75 J/m^2^ UV, but *only* for the Δ*tolC* mutant (compare gray lines in Figure 3A,B). This clearly demonstrates that ATBC is a substrate for the TolC efflux pump system [32]. Due to this increased sensitivity for ATBC, all subsequent studies were performed in the Δ*tolC* mutant strain.

We repeated these growth assays using 4-NQO. At the sub-MIC concentration of 10 µM, 4-NQO only slightly delays the midpoint of growth (∼1.5 h, Figure 3C, dashed line); however in conjunction with 5 µM ATBC, a substantial reduction (>6 h) in growth was observed (Figure 3C, gray line). Furthermore, complete growth inhibition was observed when ATBC was used at 10 µM (Figure 3D, gray line).

### Locating ATBC-binding sites on UvrA using *in silico* modeling

**Figure 4:**
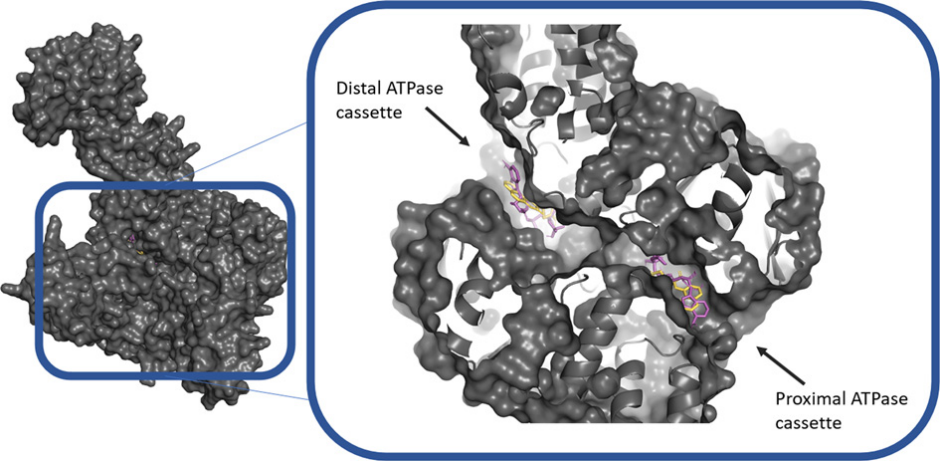
*In silico* docking of ATP and ATBC with UvrA UvrA (α-fold) is shown with ATP (magenta) and ATBC (yellow) docked in the ATP-binding pockets. The left image shows the full protein (only a monomer was used for docking); and on the right, the same image is zoomed onto the channel connecting the two ATPase domains and made visible through slab transparency. The docking was repeated six-times to check for reproducibility.

We have confirmed that ATBC affects NER using the Δ*tolC* strain. Therefore, we hypothesized that the target for ATBC could lie in UvrA, the damage-locating protein in NER. Unlike traditional docking, where the aim is to search for an optimal interaction between a ligand and target, we sought to discover all possible binding sites for ATBC on UvrA. The docking algorithm (AutoDock Vina) uses an unrestricted stochastic docking approach to find the best conformation (with the tightest binding affinity), after a random perturbation of the chemical’s flexible bonds is performed [21]. We trialled the approach using ATP, and the algorithm successfully identified both of the ATPase cassettes as binding pockets, with an average affinity of -9.48 kcal/mol (SEM = 0.07, *n* = 6). When ATBC was docked with the same parameters, the software placed the binding site in the same cavities as ATP (Figure 4), and with a comparable average binding affinity of -9.53 kcal/mol (SEM = 0.17, *n* = 6).

### Validating *in silico* modeling by assessing ATPase activity

**Figure 5:**
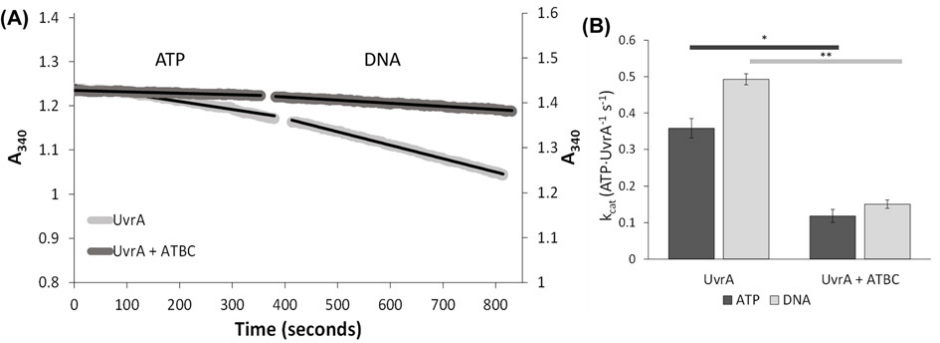
ATBC inhibits the *E. coli* UvrA ATPase activity A real-time NADH-coupled ATPase assay was performed by adding 1 mM ATP (saturating ATP [8]) to start the reaction followed by 50 ng of pUC18 DNA (indicated as a break in the trace). (**A**) Example traces clearly show that ATBC reduces the rate of ATP consumption by UvrA. The slopes provide the *V*_max_ ATP consumption rate. (**B**) *k*_cat_ values were calculated from the known concentration of UvrA. *k*_cat_ values in the presence of ATP only are shown in dark bars, and with both ATP and DNA in light bars. ATBC reduces the observed k_cat_ by ∼70%. Experiments were performed at room temperature in the ABC buffer, no ADP accumulates in this assay. The error bars represent the standard error of the mean (UvrA *n* = 26, UvrA + ATBC *n* = 6). *P = 0.0002, **P < 0.0001 (unpaired *t*-test).

The *in silico* results clearly identified ATBC’s binding to UvrA’s ATP-binding pocket, therefore, we reasoned that this would interfere with its ATPase activity. We analyzed the ATPase activity of purified recombinant UvrA when treated with ATBC in an NADH-linked ATPase assay. Figure 5A shows the loss of absorbance at OD_340_ associated with the conversion of NADH/H^+^ to NAD^+^; this absorbance change is 1:1 proportional with the conversion of ATP to ADP. The difference in rate of ATP hydrolysis in the presence and absence of 50 µM ATBC is clear in a raw data trace, shown in Figure 5A, where a reduced slope indicates reduced UvrA ATPase. ATBC led to ∼70% reduction in UvrA’s *k_cat_* (0.151 ± 0.012 s^−1^) compared with the untreated control (0.493 ± 0.015 s^−1^). As shown in Figure 5B, these differences are statistically significant. We used considerably higher concentrations of ATBC in *in vitro* assays to ensure saturation of ATBC in the aqueous solution, to offset its reduced solubility.

### Directly visualizing the effects of ATBC on UvrA’s ability to load onto DNA

**Figure 6:**
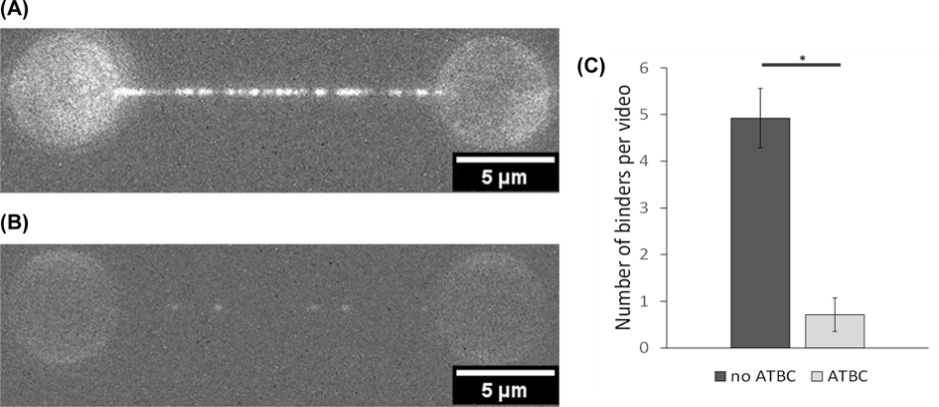
ATBC reduces the ability of UvrA to bind DNA (**A**) Five nmole UvrA-mNeonGreen binds extensively to DNA suspended between optically trapped beads. (**B**) In the presence of 50 µM ATBC, a strong reduction in UvrA-mNeonGreen binding is seen. Counting of the number of bound UvrA-mNeonGreen molecules enabled binding to be quantified. (**C**) The graph shows the average number of binders recorded in 200 frames (46.5 s). *n*_UvrA alone_ = 13 (three strands). *n*_ATBC_ = 7 (five strands). *P = 0.0002 (unpaired *t*-test). This shows an approximately sevenfold decrease in binding in the presence of ATBC. All experiments were performed in ABC buffer supplemented with 1 mM ATP and 1 mM DTT.

We have above shown that DNA stimulates the ATPase activity of UvrA and that the attached lifetime of UvrA on damaged DNA is increased by two- to threefold in the presence of ATP [8]. To determine if ATBC affects UvrA’s DNA binding, we adopted the approach of single-molecule imaging. Using lambda-phage DNA as our DNA substrate tagged at each end with biotin, we suspended this DNA between two 4.8 µm diameter streptavidin beads captured in optical traps. Five nmole UvrA-mNeonGreen was flowed into the experimental chamber and incubated for 2 min, in the presence or absence of 50 µM ATBC. In the absence of ATBC, the DNA tether was completely coated with UvrA-mNeonGreen (Figure 6A). However, in the presence of 50 µM ATBC, the extent of binding was severely diminished and only occasional binders were observed (Figure 6B). Due to the ATBC’s propensity to form precipitates, we adapted the workflow to quantify this reduction in binding by counting individual binders. Using this approach, we estimated that the presence of ATBC reduced the binding of UvrA to DNA by approximately sevenfold (Figure 6C).

## Discussion

Exposure of bacteria to UV can lead to DNA damage, which affects replication and transcription. To maintain genomic integrity such lesions are repaired by the NER pathway. In addition, this pathway plays a role in repairing the damage caused by radical oxygen species, which are generated during the host immune response to infection [6,7,33,34]. Therefore, this pathway that has potential as an antimicrobial target has been underexplored. In the present study, we determine the mechanism and structural mode of action for the only known inhibitor of bacterial NER, ATBC [14], and assess its utility as an antimicrobial agent as a step toward the development of future compounds aimed at a new protein target.

### ATBC as an antimicrobial agent

ATBC was found to be nontoxic even at very high concentrations (MIC > 125 µM), however, was very effective at inhibiting *E. coli* growth after exposure to DNA-damaging agents. Both UV irradiation and cellular treatment with the UV mimic, 4-NQO, led to significant reductions in cell growth in the presence of ATBC. Together, these are ideal properties for an inhibitor that is designed to work in combination with other agents to improve the therapeutic profiles of otherwise less effective drugs. An example of such adjuvant activity in antimicrobial development is the addition of clavulanic acid to offset the resistance to Penicillin due to β-lactamase activity [35].

Despite the positive aspects of ATBC, its poor solubility in water makes it difficult to estimate real concentrations and impairs experimental reproducibility. Especially problematic are longer incubations or treatments at high compound concentrations. Furthermore, poor solubility can impair the bioavailability, the administration route and even cause toxicity. This is a major problem encountered in drug discovery that can be tackled by medicinal chemists in multiple ways [36,37]. An example is the addition of polar groups to Tioconazole, a poorly soluble antifungal agent used for skin infection, to create Fluconazole, an antifungal with improved solubility that facilitates systemic use [38]. Finally, we also determined that ATBC is a TolC substrate, therefore, clinical strains will most likely have a diminished sensitivity to the compound [39].

### ATBC inhibits NER through interaction with UvrA

Computational approaches for studying ligand binding to proteins offer an unbiased method for determining how inhibitors might work at a molecular level. Molecular docking is a widely used tool with multiple applications in drug discovery, from virtual screening to structure–activity studies [40,41]. Here, using AutoDock Vina, we determined that ATBC binds to both of UvrA’s nucleotide-binding pockets. When exploring the possible binding sites, we did not aim to find the best docking conformation, instead by focusing on the most targeted location we obtained an indication of how the inhibitor might work. These findings were supported by ATPase results that indicated ATBC reduces UvrA’s ATPase activity over threefold. Notably, the *k*_cat_ was not brought to zero at this concentration of ATBC, which may suggest incomplete saturation consistent with a competitive mode of action, or a preferential binding to one ATPase in respect of the other. Unfortunately, ATBC’s poor solubility makes a determination of *K*_i_ impossible. However, using a single-molecule imaging approach, we were able to show a profound reduction in binding of UvrA to DNA in the presence of 50 µM ATBC. The incomplete loss of binding inhibition was consistent with a reduction and not complete loss of UvrA’s ATPase. This links UvrA’s ATPase sites to its ability to bind DNA, such a low-affinity ATPase state was shown to be the ADP-bound state [8] Therefore, it is possible that ATBC binding mimics the ADP-bound state of UvrA.

UvrA-binding DNA is essential for DNA repair by NER, since UvrA locates damage prior to loading UvrB, which verifies its presence before initiating excision repair [7]. The process of recognition involves multiphase kinetic proofreading, an initial rapid contact with damaged DNA by UvrA does not consume ATP (Charman and Kad, personal communication); however, UvrA uses ATP to verify the presence of damage [42,43]. With UvrA compromised by bound ATBC, it cannot successfully verify damage effectively shutting down the NER system. Therefore, it is clear why its inhibition represents a target for drug development as part of a combinatorial therapy. Despite being an effective inhibitor, ATBC faces substantial problems due to its poor solubility and susceptibility to TolC. Therefore, this molecule should be seen as a template for a new class of inhibitors through the generation of more active analogs with an enhanced chemical profile as above for numerous other compounds [44,45].

In summary, we have shown that ATBC is a TolC substrate active against *E. coli* following DNA damage as previously shown for *M. tuberculosis*. The present study also demonstrates the powerful combination of computational docking and subsequent biochemical assays for determining the mechanism of inhibitor action. For ATBC, we find that it likely acts as an ADP analog and using single-molecule imaging we confirm that it reduces UvrA’s binding to DNA. Altogether, we have not only determined the mechanism of action for ATBC, but we have also highlighted its lack of solubility and propensity to be removed by TolC as a major drawback for development as an inhibitor. The promise of this compound for use as a new antimicrobial is clear, however, its physical properties need to be improved through chemical modification.

## Supplementary Material

Supplementary Figure S1

## Data Availability

All data produced is available upon request.

## Open Access

Open access for the present article was enabled by the participation of University of Kent in an all-inclusive *Read & Publish* agreement with Portland Press and the Biochemical Society under a transformative agreement with JISC.

## Abbreviations

4-NQO: 4-nitroquinoline 1-oxide
AMR: antimicrobial resistance
ATBC: 2-(5-Amino-1,3,4-thiadiazol-2-yl)benzo(f)chromen-3-one
CLSI: Clinical and Laboratory Standards Institute
MIC: minimum inhibitory concentration
NER: nucleotide excision repair
PMSF: phenylmethylsulfonyl fluoride
