# Supplementary material for: Identification of the target and mode of action for the prokaryotic nucleotide excision repair inhibitor ATBC
Source: Biosci Rep. 2022 May 30;42(6):BSR20220403. doi: 10.1042/BSR20220403 (PMC9152706; doi:10.1042/BSR20220403)
Supplement: Supplementary Figure S1 [file BSR-2022-0403_supp.pdf]

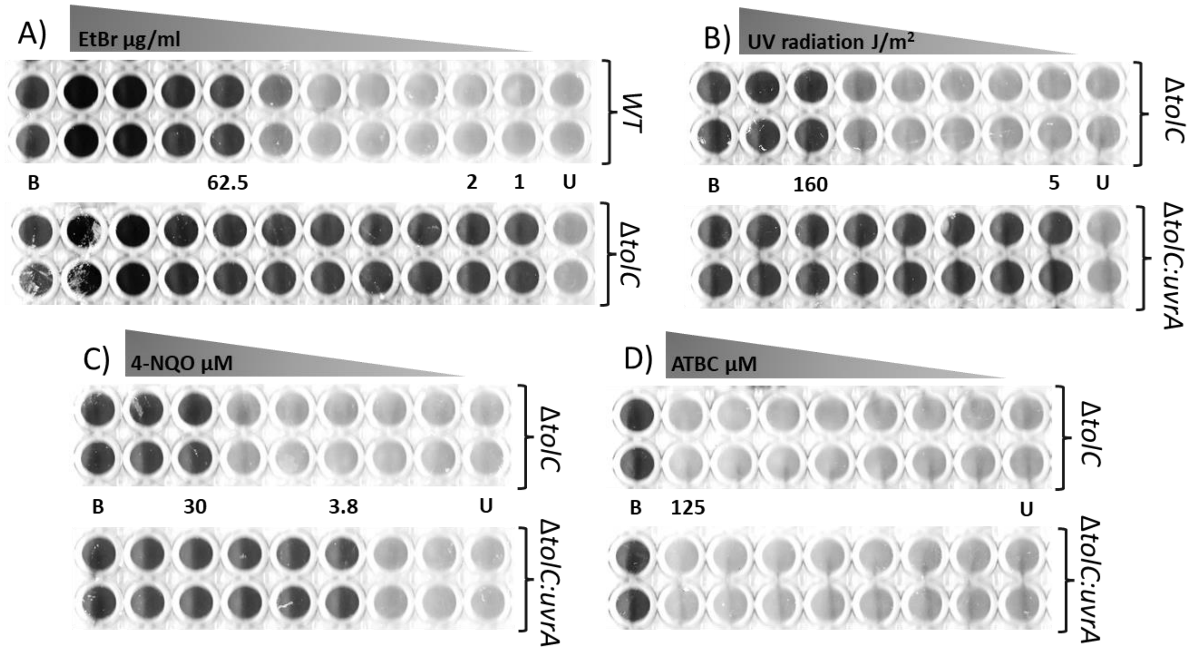

A)
EtBr µg/ml
WT
B
62.5
2
1
U
ΔtolC
B)
UV radiation J/m2
ΔtolC
B
160
5
U
ΔtolC:uvrA
C)
4-NQO µM
ΔtolC
B
30
3.8
U
ΔtolC:uvrA
D)
ATBC µM
ΔtolC
B
125
U
ΔtolC:uvrA

Figure S1: Determining minimal inhibitory concentrations (MIC). *After 20 h incubation of cells at 37°C (16h incubation prior to the addition of resazurin and further incubation for 4h), the blue color shows no growth, whereas the pink wells represent growing cells (B = MOPS minimal media as sterility control, U = untreated cells). Numbers refer to concentrations or exposure as indicated. A) The EtBr MIC shows a ≈ 64-fold increase in sensitivity when the TolC pump is knocked out. B) UV irradiation MIC, shows a ≈ 32-fold increase in sensitivity to UV with the uvrA knockout. C) 4-NQO MIC shows a 30 µM MIC for the compound which decreased 8-fold in the uvrA knockout, confirming the compound to be a substrate for NER. D) ATBC MIC, the MIC is > 125 µM for both strains used, indicating ATBC is non-toxic. The experiments were performed in triplicate on three independent days. No appreciable difference was identified among the replicates. This is a black and white reproduction of Figure 2.*
